# Deep Learning for Automatic Diagnosis and Morphologic Characterization of Malignant Biliary Strictures Using Digital Cholangioscopy: A Multicentric Study

**DOI:** 10.3390/cancers15194827

**Published:** 2023-10-01

**Authors:** Miguel Mascarenhas Saraiva, Tiago Ribeiro, Mariano González-Haba, Belén Agudo Castillo, João P. S. Ferreira, Filipe Vilas Boas, João Afonso, Francisco Mendes, Miguel Martins, Pedro Cardoso, Pedro Pereira, Guilherme Macedo

**Affiliations:** 1 Department of Gastroenterology, São João University Hospital, Alameda Professor Hernâni Monteiro, 4200-427 Porto, Portugal; 2 WGO Gastroenterology and Hepatology Training Center, 4200-319 Porto, Portugal; 3 Faculty of Medicine, University of Porto, Alameda Professor Hernâni Monteiro, 4200-427 Porto, Portugal; 4 Department of Gastroenterology, Hospital Universitario Puerta de Hierro Majadahonda, C/Joaquín Rodrigo, 28220 Majadahonda, Madrid, Spain; 5 Department of Mechanical Engineering, Faculty of Engineering, University of Porto, Rua Dr. Roberto Frias, 4200-465 Porto, Portugal; 6 DigestAID—Digestive Artificial Intelligence Development, Rua Alfredo Allen n.º 455/461, 4200-135 Porto, Portugal

**Keywords:** cholangioscopy, artificial intelligence, biliary strictures

## Abstract

**Simple Summary:**

Diagnosis and characterization of biliary strictures is challenging, even after the introduction of digital single-operator cholangioscopy (D-SOC). The endoscopist’s visual impression has a suboptimal accuracy and there is a significant interobserver variability. Artificial intelligence tools for image analysis have presented important contributions in several fields of gastroenterology. Convolutional neural networks are highly efficient multi-layered deep neural networks for image analysis, with great results in several fields of medicine. Nevertheless, the role of these deep learning models in digital cholangioscopy is still in a premature phase. With this bicentric international study, the authors aimed to create a deep learning-based algorithm for digital cholangioscopy capable of distinguishing benign from malignant biliary lesions. The present model accurately detected malignant biliary lesions with an image processing rate that favors its clinical applicability. The authors believe that the use of an AI-based model may change the landscape in the digital cholangioscopy diagnostic yield.

**Abstract:**

Digital single-operator cholangioscopy (D-SOC) has enhanced the ability to diagnose indeterminate biliary strictures (BSs). Pilot studies using artificial intelligence (AI) models in D-SOC demonstrated promising results. Our group aimed to develop a convolutional neural network (CNN) for the identification and morphological characterization of malignant BSs in D-SOC. A total of 84,994 images from 129 D-SOC exams in two centers (Portugal and Spain) were used for developing the CNN. Each image was categorized as either a normal/benign finding or as malignant lesion (the latter dependent on histopathological results). Additionally, the CNN was evaluated for the detection of morphologic features, including tumor vessels and papillary projections. The complete dataset was divided into training and validation datasets. The model was evaluated through its sensitivity, specificity, positive and negative predictive values, accuracy and area under the receiver-operating characteristic and precision-recall curves (AUROC and AUPRC, respectively). The model achieved a 82.9% overall accuracy, 83.5% sensitivity and 82.4% specificity, with an AUROC and AUPRC of 0.92 and 0.93, respectively. The developed CNN successfully distinguished benign findings from malignant BSs. The development and application of AI tools to D-SOC has the potential to significantly augment the diagnostic yield of this exam for identifying malignant strictures.

## 1. Introduction

Biliary strictures (BSs) are a concerning finding, often confronting patients with a poor prognosis. The primary focus in the presence of a BS is to exclude malignancy, which is responsible for the majority of BS cases. Malignant BSs typically result from primary (cholangiocarcinoma) or secondary neoplasia with biliary tract extension (gallbladder, pancreatic, hepatocellular carcinoma) [1,2]. On the other hand, around 30% of all BS cases are benign, with the need to consider iatrogenic causes, biliary lithiasis, primary and IgG4-related sclerosing cholangitis [2,3]. Therefore, it is crucial to differentiate between benign and malign BSs, as the treatment and prognosis greatly differ between the different etiologies [4,5].

Endoscopic retrograde cholangiopancreatography (ERCP) has historically been the primary diagnostic modality in patients with biliary strictures. This technique allows the observation of indirect signs that may suggest the malignant nature of BSs (surface irregularity, stricture length), together with tissue sampling, either by brush cytology or fluoroscopy-guided transpapillary biopsy. However, the diagnostic performance of ERCP combined with brush cytology or biopsy is poor [6]. Indeed, a meta-analysis reported a sensitivity of 45% for brush cytology and 48% for ERCP-guided biopsies. The combination of both methods modestly increased the sensitivity for detection of malignant biliary strictures [7].

Digital single-operator cholangioscopy (D-SOC) allows high-resolution inspection of the bile duct, enabling its application for diagnostic and therapeutic purposes. Direct visualization allows for more accurate morphologic characterization of BSs as well as the possibility for targeted biopsies [8]. A recent multicentric randomized trial demonstrated the higher sensitivity of D-SOC for the visual identification of malignant strictures, compared to standard ERCP cholangiographic impression (96% vs. 67%, *p* = 0.02) [9]. Nonetheless, the specificity of the visual impression remains suboptimal (89%) [10]. In fact, the accuracy is diminished when evaluating extrinsic BSs (most commonly pancreatic adenocarcinoma or metastatic disease) [11]. Additionally, the presence of traumatic lesions after stent removal or even the passage of the scope may be mistaken with malignant lesions. Lastly, the presence of diseases associated with chronic biliary duct inflammation (namely primary sclerosing cholangitis) is associated with a decreased diagnostic yield for diagnosing malignant BSs.

Several morphological features are associated with an increased malignancy risk [12,13]. The identification of papillary projections is associated with a seven-times increased risk of malignancy in a multivariate analysis [14]. Nevertheless, a significant lack of interobserver agreement in this morphologic feature identification was observed. On the other hand, abnormal dilated tumor vessels are commonly visualized in malignant BSs [12]. These vessels are developed during tumoral angiogenesis and are associated with an accurate detection of malignant BSs [15]. However, chronic inflammation can diminish the diagnostic accuracy of D-SOC for tumoral vessels. Therefore, classification systems for prediction of BS malignancy have been tested, namely systems based on morphological features [14,16]. Sethi et al. developed the Monaco Classification System for indeterminate BSs, reporting an overall accuracy of 70% for malignant BSs and relevant interobserver agreement for papillary projection (k = 0.43) and abnormal vessels identification (k = 0.26) [14]. However, there is no universally accepted classification system for D-SOC findings and interobserver agreement between different endoscopists remains poor [17].

The development of artificial intelligence (AI) models suited for the analysis of large image datasets is a matter of great scientific interest, specially using deep learning algorithms. Convolutional neural networks (CNN) are a human visual cortex inspired multilayered deep learning model suitable to increase the diagnostic yield in several medical fields [18,19,20]. Additionally, there are several published studies about the impact of these models in the diagnostic performance of several endoscopic techniques [21,22,23].The impact of AI for the evaluation of cholangioscopy images has recently started to be investigated. However, a tool providing categorization (i.e., discriminating malignant from non-malignant strictures) as well as morphologic classification has scarcely been assessed. Given the current limitations in the diagnostic approach to biliary strictures and the potential of AI to provide effective image analysis, our group aimed to develop and validate a CNN model for automatic detection and differentiation between benign and malignant BSs in D-SOC. Additionally, our group assessed the capacity of the CNN to provide accurate identification of significant morphological features of malignant BSs.

## 2. Materials and Methods

### 2.1. Patient Population and Study Design

For the development of the study, our group included D-SOC exams performed between August 2017 and November 2022 at two centers in Portugal (Centro Hospitalar Universitário de São João (CHUSJ), Porto, Portugal) and Spain (Hospital Universitario Puerta de Hierro Majadahonda (HUPHM), Madrid, Spain). A total of 124 patients (CHUSJ, n = 106; HUPHM, n = 18), corresponding to 129 D-SOC exams (CHUSJ, n = 111; HUPHM, n = 18), were enrolled. A total of 84,994 still-frame images were used for the development, training and validation phases of the CNN for automatic differentiation between malignant and benign BSs. The still-frame images were obtained during the exam, mainly through decomposition of the procedure videos into frames, using a VLC media player (VideoLAN, Paris, France).

The study was performed after approval by the ethics committee of Centro Hospitalar Universitário de São João/Faculdade de Medicina da Universidade do Porto (CE 41/2021) and Hospital Universitario Puerta de Hierro Majadahonda (PI 153/22). This was a retrospective non-interventional study, performed with respect for the Declaration of Helsinki. An adequate omission of potentially identifiable patient information was assured, with each individual patient being assigned with a random number, guaranteeing data anonymization. The non-traceability of the data and respect to the general data protection regulation (GDPR) was assured by a team with a Data Protection Officer (DPO).

### 2.2. Digital-Single Operator Cholangioscopy Procedure and Definitions

All of the D-SOC exams included in the study were performed with the SpyGlass™ DS II system (Boston Scientific Corp., Marlborough, MA, USA). The procedures were performed by expert gastroenterologists (P.P., F.V.B., M.G.-H., and B.A.G), each with experience of more than 2000 ERCPs and 100 cholangioscopies prior to this study. For the performance of the exams the Olympus TJF-160V or TFJ-Q180V duodenoscopes (Olympus Medical Systems, Tokyo, Japan) were used. Additionally, the SpyBite^TM^ forceps (Boston Scientific Corp., Marlborough, MA, USA) were utilized for obtaining the biopsy specimens with direct visual guidance, assuring a minimum of 4 biopsies in all the study exams.

A total of 84,994 D-SOC biliary images were classified as benign or malignant. Benign biliary findings typically included normal bile ducts, stone disease and benign BSs. A benign BS-confirmed diagnosis implied a negative histopathology (biopsy or surgically obtained) with absence of malignancy after 6 months of follow up. Stone disease was diagnosed upon direct observation and in the absence of other findings. A malignant diagnosis implied a malign histopathology, obtained either through D-SOC biopsy or other tissue sampling exams (namely brush cytology, fluoroscopic or endoscopic ultrasound-guided biopsy or even surgical specimen).

### 2.3. Development of the Convolutional Neural Network

We developed a deep learning-based CNN to automatically detect and differentiate malignant biliary strictures from benign biliary conditions, the latter including benign strictures, stone disease and normal bile ducts. A total of 84,994 frames were included: malignant strictures were seen in 44,743 images; whereas the remaining 40,521 showed benign biliary conditions.

**Figure 1:**
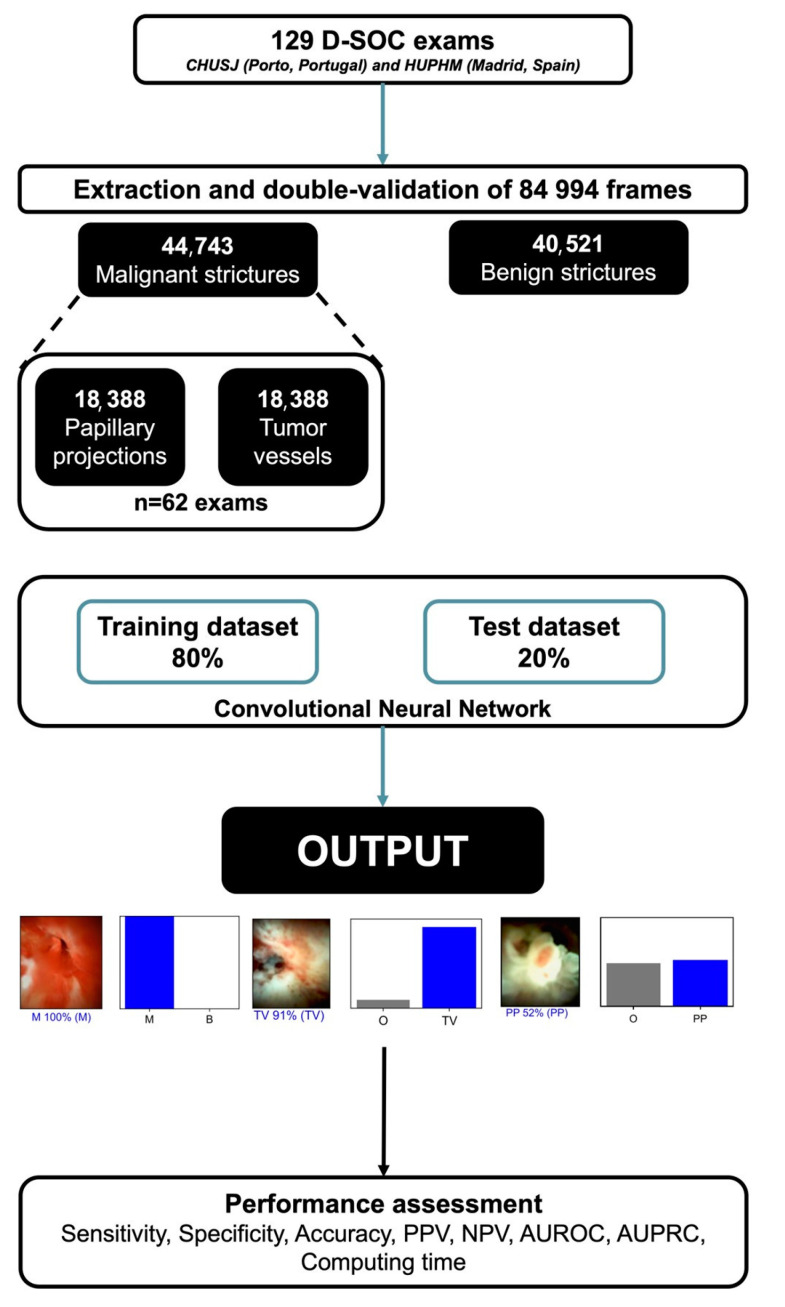
Study flowchart for the training and validation phases. AUC—area under the receiving operator characteristic curve; B—benign biliary findings; D-SOC—digital single-operator cholangioscopy; M—malignant stricture; NPV—negative predictive value; PP—papillary projections; O—other; PPV—positive predictive value; TV—tumor vessels.

The total data was separated into two sets: training and validation. The first comprised 80% of the frames (n = 67,678), while the second used 20% of the remaining images (n = 17,316) using a patient-split design, ensuring that no data from the same patient overlapped in both the datasets. The validation dataset was used to assess the model’s performance. A graphical flowchart of the study design is shown in Figure 1. Additionally, in a subset of exams (n = 62) we evaluated the performance of the CNN for the detection of morphologic features associated with bile duct malignancy (“tumor vessels” and “papillary projections”). Tumor vessels were defined as abnormal, dilated, tortuous vessels associated with bile duct malignancy (n = 18,388). Papillary projections (n = 18,388) were represented as finger-like projections associated with bile duct malignancy.

The Resnet model was used to build this CNN. ImageNet, a large-scale collection of images used for object recognition software development, was used to train weights between units. We preserved its convolutional layers to impart its learning to our model. The final fully connected layers were deleted and replaced with new fully connected layers according to the number of classes we used to categorize our endoscopic frames. There was an initial fully connected layer in each of the two blows that we used, followed by dropout layers with a drop rate of 0.1. After that, we added a dense layer whose size defined the number of classification groups (two: malignant strictures and benign biliary conditions). The learning rate was 0.00015, the batch size was 128 and the number of epochs was 10. PyTorch was used to prepare and run the model. Performance analyses was carried out with a computer equipped with a 2.1 GHz Intel^®^ Xeon^®^ Gold 6130 processor (Intel, Santa Clara, CA, USA) and a double NVIDIA Quadro^®^ RTX™ 4000 graphic processing unit (NVIDIA Corporate, Santa Clara, CA, USA).

### 2.4. Model Performance and Statistical Analysis

**Figure 2:**
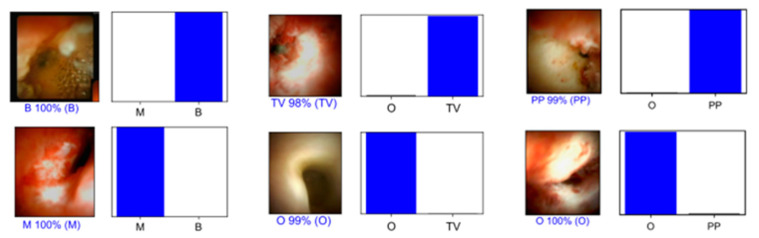
Output obtained during the training and development of the convolutional neural network. The bars represent the probability estimated by the network. The finding with the highest probability was output as the predicted classification. A blue bar represents a correct prediction. B—benign biliary findings; M—malignant stricture; O—other; PP—papillary projections; PPV—positive predictive value; TV—tumor vessels.

The assessment of CNN’s performance was performed using an independent validation dataset (20% of all the data). For each frame, the algorithm calculated the probability of having a malignant stricture and the probability of being considered a benign biliary condition (Figure 2). Since a higher probability translated into greater confidence of the CNN prediction, the model selected the category with the highest probability as its final classification. Then, the final classification of the CNN was compared with the corresponding histopathological evaluation, which was regarded as the gold standard. Sensitivity, specificity, positive predictive value (PPV), negative predictive value (NPV) and accuracy in distinguishing malign strictures from benign biliary conditions were our primary outcomes. Additionally, we performed receiver operating characteristic (ROC) curves analysis and calculated the area under the ROC curves (AUROC) to evaluate the discriminatory capacity of our model. Moreover, the precision-recall (PR) curve and the area under the precision-recall curve (AUPRC) were used to measure the performance of the model, accounting for potential data imbalance. Finally, we evaluated the computational performance of the algorithm by measuring the time required for the CNN to process and generate output for all the frames included in the validation dataset. We performed statistical analysis using Sci-Kit learn v0.22.2 [24].

## 3. Results

### 3.1. Performance of the Convolutional Neural Network

In total, 129 D-SOC exams were performed in 124 patients, from August 2017 to November 2022. In 73 patients, a diagnosis of malignancy was established. Benign findings were established in 51 patients. We included 84,994 frames for development of this CNN, of which 44 743 were malignant strictures. The remaining 40,521 images were benign biliary conditions (benign strictures, stone disease and normal bile ducts).

**Figure 3:**
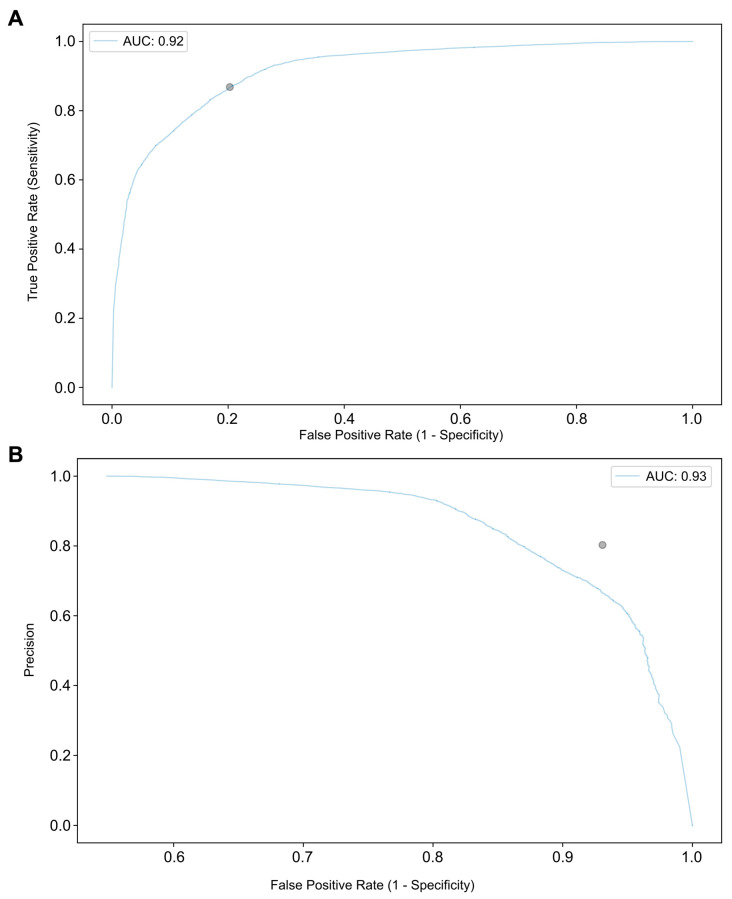
Receiver operating characteristic (**A**) and precision-recall (**B**) analyses of the network’s performance in the detection of malignant biliary strictures or benign biliary conditions.

The model was trained and developed using 80% of the total dataset (n = 67,678). The remaining 20% (n = 17,316) was used to test the algorithm’s performance. Table 1 shows the confusion matrix between the CNN’s predictions in validation set versus the histopathologic characterization. In terms of detecting and distinguishing malign strictures from benign conditions, the CNN was associated with a sensitivity of 83.5%, a specificity of 82.4% and an accuracy of 82.9%. PPV and NPV were, 79.6% and 85.8%, respectively. The model’s AUROC and AUPRC for differentiating between the malignant lesions and benign biliary conditions were 0.92 and 0.93, respectively, as shown in Figure 3.

### 3.2. Detection of Morphological Characteristics Associated with Biliary Malignancy

**Figure 4:**
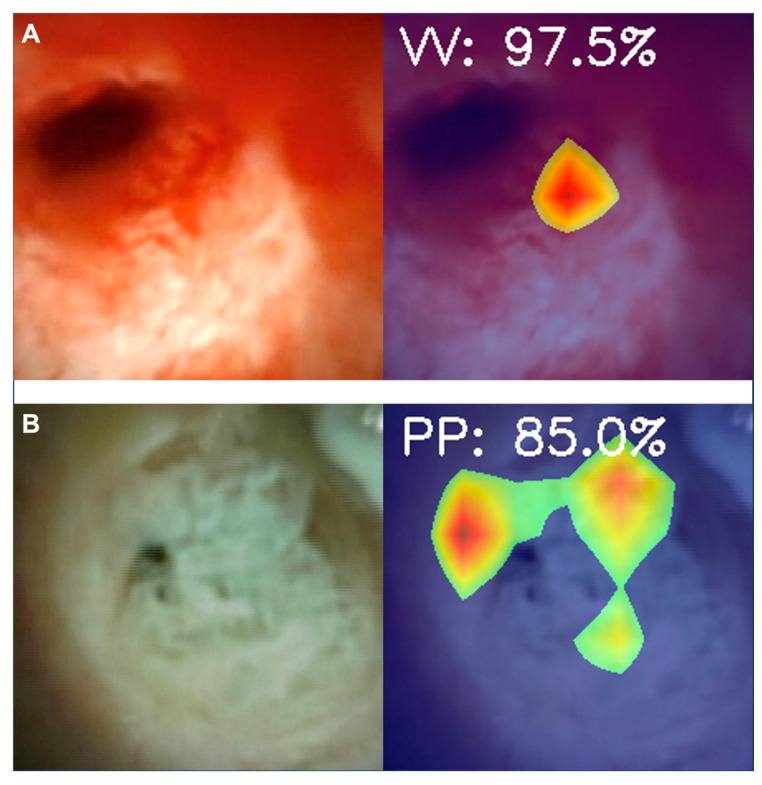
Heatmap analysis showing the prediction of the algorithm for the identification of tumor vessels (**A**) and papillary projections (PP), with the associated probability. (**A**)—Tumor vessels (VV); (**B**)—Papillary projections (PP).

**Figure 5:**
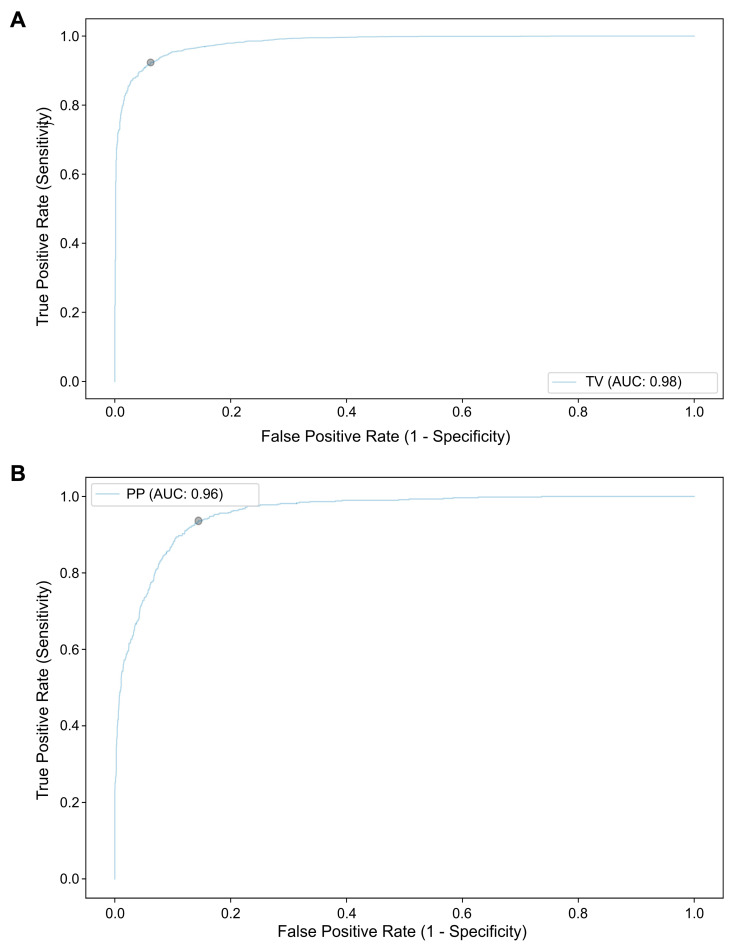
ROC analysis of the network’s performance in the detection of morphological characteristics of malignancy. (**A**)—Tumor vessels (TV); (**B**)—Papillary projections (PP).

The CNN’s performance for the detection of morphological features associated with malignancy of the biliary tract (tumor vessels and papillary projections) were also assessed on a subset of 62 D-SOC exams of patients with malignant biliary strictures. Two sets of 18,388 images were used for the constitution of the CNNs for the automatic detection of TV and PP, respectively. Heatmap analysis was performed for the identification of features contributing to the predictions of the CNN (Figure 4). Regarding tumor vessel detection, the CNN sensitivity and specificity were 95.7% and 88.6%, respectively, with an accuracy of 93.0%. In terms of papillary projection identification, the model’s sensitivity, specificity and accuracy were 74.1%, 94.5% and 91.2%, respectively. The AUROC for the detection and differentiation of tumor vessels and papillary projections by the CNN was 0.98 and 0.96, respectively (Figure 5).

### 3.3. Computational Performance of the CNN

The CNN processed 4250 batches (each batch comprising of 128 frames) in 23 min and 16 s, which can be translated to an approximate reading rate of 390 frames per second.

## 4. Discussion

The utilization of AI tools in medical routines is experiencing rapid growth. Research in the field of deep learning systems in the field of gastrointestinal endoscopy has primarily concentrated on luminal endoscopy, while the research on hepatobiliary indications is significantly less robust [25]. Obtaining a conclusive diagnosis in patients with indeterminate bile duct strictures is crucial for tailoring treatments for each patient. Nonetheless, it is often challenging to attain a specific diagnosis due to frequently inconclusive tissue sampling. Recently, Gerges et al. demonstrated a higher sensitivity of D-SOC-guided biopsies compared to those obtained during ERCP procedures [9]. The sensitivity of D-SOC-guided biopsies was calculated at 74% in a recent meta-analysis [26]. Nevertheless, the introduction of D-SOC brought about a remarkable improvement, particularly evident in the accuracy of visually assessing significant biliary lesions. In a study conducted by Navaneethan et al., the estimated sensitivity for visual impression in diagnosing malignancy was reported to be an impressive 90% [27]. However, the diagnosis of malignancy through visual impression alone is hindered by suboptimal specificity and accuracy [14,28]. Currently, a universally accepted classification system for visually diagnosing malignancy during single-operator cholangioscopy is yet to be clinically established [12,13,14]. Furthermore, the utilization of existing classification systems has been linked to inadequate interobserver agreement, exacerbating the challenges in this field [14].

The primary objective in managing a biliary stricture is to effectively exclude malignancy. The advent of D-SOC has notably improved the diagnostic accuracy for indeterminate biliary strictures. Nonetheless, a missing rate as high as 10% has been reported for D-SOC with direct visualization or targeted biopsies [10], and a definite diagnosis of malignancy imperatively requires histologic confirmation. Considering these constraints, we firmly believe that integrating real-time AI technology into D-SOC has the potential to bridge this gap and address these challenges effectively. A recent systematic review and meta-analysis by Njei et al. suggested the application of AI systems as the most promising solution for the distinction between malignant and benign BSs [29]. Recently, significant interest has been devoted to AI algorithms for the identification of malignant biliary strictures. Robles-Medranda et al. developed a CNN-based model for the identification of biliary malignancy using pre-defined endoscopic classifications [30]. This algorithm has been shown to be highly accurate in the detection of tumor vessels. Moreover, the CNN outperformed non-expert endoscopists in the identification of malignant BSs. Nevertheless, this study has not performed explainability analysis, thus not allowing the full assessment of the predictions of the CNN. More recently, Zhang et al. have developed consecutive deep learning algorithms for the selection of quality D-SOC images for subsequent development of a CNN for the classification of biliary strictures [31]. Their deep learning algorithm achieved a sensitivity of 92% and a specificity of 88% for the detection of malignant strictures at a video level. This study simultaneously provided heatmap analysis to ascertain suspicious areas, which allowed the identification of areas contributing significantly for the predictions of the algorithm. The improvement in the accuracy of AI systems integrated in real-time into D-SOC systems may enhance the evaluation of visual features of biliary strictures. However, it is crucial to note that these systems are expected to assist instead of replacing conventional tissue sampling. Integrating visual features linked to a higher likelihood of malignancy (such as tumor vessels and papillary projections) into these models can facilitate the precise identification of areas where suspected malignant lesions are present. This, in turn, has the potential to enhance the diagnostic yield of existing D-SOC-guided tissue sampling. Further development of these algorithms, combined with ongoing efforts to improve staging and prognostication with the assistance of AI, will provide more personalized care to patients with suspected biliary malignancy, thus offering the potential to improve the prognosis of these patients [32,33,34,35].

The algorithm developed in this study had a dual purpose: to categorize biliary strictures as either malignant or benign and to identify the morphological features associated with an increased risk of malignancy, such as tumor vessels and papillary projections. To ensure a robust and diverse dataset, we included images from two large-volume centers, resulting in a comprehensive collection of nearly 85,000 biliary stricture images. The findings of our study revealed that this model demonstrated exceptional sensitivity, specificity and accuracy. Overall, our network achieved an AUC of 0.92 in distinguishing malignant from benign strictures. Additionally, our CNN exhibited outstanding performance in detecting tumor vessels and papillary projections, with AUC values of 0.98 and 0.96, respectively. Furthermore, our algorithm displayed remarkable image processing efficiency, with an approximate reading rate of 390 frames per second. Our results are in line with a recent study by Marya et al., which focused on the development of a CNN for the identification of malignant BSs, which showed an adequate performance in differentiating malignant from benign BSs, with an overall accuracy of 91% [36]. These results demonstrate the potential of these systems in advancing the field of biliary stricture diagnosis and management.

This study has several points of merit. First, the development of this deep learning algorithm included a large volume and variety of images obtained from D-SOC exams performed at two European high-volume referral centers. Second, we included a robust dataset of almost 85,000 images of patients with BSs, for whom the diagnosis of biliary malignancy required unequivocal histological proof. Third, we have built upon previously published work on the application of AI systems to D-SOC. In this study, we have expanded the evidence on the application of these algorithms for the detection of morphological features associated with an increased risk of biliary malignancy. Indeed, our system detected tumor vessels and papillary projections with an AUC of 0.98 and 0.96. The detection of these morphologic features is of paramount importance as they have been demonstrated to predict the presence of malignant BSs. Indeed, Robles-Medranda and coworkers have shown that the identification of tumor vessels predicted the presence of a malignant biliary stricture with a sensitivity of 94% and an overall accuracy of 86%. Nevertheless, the specificity of this finding was suboptimal (63%) [15]. The introduction of AI models may provide a solution in decreasing the problematic issues of both false negative and false positive results, which lead to inadequate treatments and morbidity. Indeed, our network achieved a sensitivity of 96% and a specificity of 87%. This is in line with previously published studies on the detection of tumor vessels in malignant BSs [30,37]. Besides the importance of classifying a BS as malignant or benign, real-time AI models accurately identifying the morphologic features associated with biliary malignancy may provide guidance to D-SOC-oriented tissue sampling, therefore increasing its yield.

This study has several limitations to be acknowledged. First, despite the large dataset for the context of a proof-of-concept study, clinical validation of this algorithm will require a much larger volume of data. Secondly, our deep learning model was developed and tested exclusively on a single D-SOC platform, which limits the generalizability of the algorithm to other cholangioscopy systems. Third, at this stage, we did not assess the use of deep learning with prior knowledge for the enhancement of the performance of our algorithm. Moreover, distinct deep learning models other than convolutional neural networks have been shown to be more efficient than CNNs [38], and their use should be assessed in further studies. Interoperability remains a significant concern in the development and application of AI technologies in the medical field, as the ability to generalize a given technology across multiple devices is a crucial requirement for its clinical applicability. Therefore, it is essential to develop and validate this deep learning model across different D-SOC devices. Thirdly, while efforts were made to mitigate the risk of overfitting, it cannot be eliminated. As other systems designed for pancreatobiliary endoscopy, the technological maturity of our algorithm remains unfit for clinical practice. Subsequent development of these algorithms on an adequate environment, as well as prototype validation in a real-life clinical setting should follow suite. Therefore, while the performance marks of this algorithm on a preclinical stage suggest that it would provide accurate predictions in a real-life setting, these results should be interpreted considering the stage of development of the algorithm. Subsequent development of these algorithms should include: the development of international multicentric studies, with the aim of increasing datasets, both in quantity as well as in variability; engaging with practitioners for the development of user-friendly prototypes, combining an increase in the accuracy provided by these software with the current standards of practice of expert centers, alerting the endoscopists for meaningful findings and preventing “noisy” overpredictions; finally, the ultimate application of these algorithms in clinical practice should be strictly regulated by competent agencies, and effective polices should be enforced to ensure the quality of these systems as well as their clinical benefit.

## 5. Conclusions

The influence of AI in everyday clinical practice is expected to continue growing in the near future. The potential impact of deep learning algorithms on the care of patients with suspected biliary malignancy is significant. This study aimed to evaluate the performance of a CNN in detecting and distinguishing between malignant and benign biliary disorders, utilizing a large dataset of D-SOC images from two experienced centers in this field. The favorable performance demonstrated by this model establishes a solid groundwork for further investigation of AI technologies in this specific patient subset, with the ultimate goal of enhancing the clinical outcomes for individuals suspected of having biliary malignancy.

## Figures and Tables

**Table 1:** Confusion matrix of the automatic detection versus final diagnosis, CNN—convolutional neural network; Malignant—malignant biliary strictures; Benign—normal bile ducts or benign biliary findings.

		Final Diagnosis	
		Malignant	Benign
**CNN classification**	Malignant	6527	1673
	Benign	1293	7823

## Data Availability

Not applicable.
